# Response to: Testing the Association Between Smoking in Pregnancy and Attention-Deficit/Hyperactivity Disorder in a Novel Design

**DOI:** 10.1016/j.biopsych.2010.01.032

**Published:** 2010-08-15

**Authors:** Anita Thapar, Frances Rice, Dale Hay, Jacky Boivin, Kate Langley, Marianne van den Bree, Michael Rutter, Gordon Harold

**Affiliations:** a Department of Psychological Medicine and Neurology, Medical Research Council Centre for Neuropsychiatric Genetics and Genomics, School of Medicine, Cardiff University, Cardiff, Wales, United Kingdom; b Department of Psychological Medicine and Neurology, Medical Research Council Centre for Neuropsychiatric Genetics and Genomics, School of Medicine, Cardiff University, Cardiff, Wales, United Kingdom; c School of Psychology, Cardiff University, Cardiff, Wales, United Kingdom; d School of Psychology, Cardiff University, Cardiff, Wales, United Kingdom; e Department of Psychological Medicine and Neurology, Medical Research Council Centre for Neuropsychiatric Genetics and Genomics, School of Medicine, Cardiff University, Cardiff, Wales, United Kingdom; f Department of Psychological Medicine and Neurology, Medical Research Council Centre for Neuropsychiatric Genetics and Genomics, School of Medicine, Cardiff University, Cardiff, Wales, United Kingdom; g SGDP Centre, Institute of Psychiatry, King's College London, London, United Kingdom; h School of Psychology, Cardiff University, Cardiff, Wales, United Kingdom

We thank the authors for their interest in our article (1). They raise a question about gender effects and highlight “it would be informative to look at the results stratified by gender.” We agree that examination of gender effects, where feasible, is important. To meaningfully examine associations between smoking in pregnancy and attention-deficit/hyperactivity disorder (ADHD) among genetically related and unrelated mother-child pairs, further stratified by child gender and exposure to smoking in pregnancy, a substantially larger sample size would be required. However, to address the points raised by Obel *et al.* (2), we provide the data stratified by gender, with the caveat that the sample size available becomes too small in the unrelated group to derive meaningful interpretations.

In the total sample, there was no significant difference in the proportion of male and female offspring of smokers versus nonsmokers. There were 377 unexposed boys, 360 unexposed girls, 29 exposed boys, and 19 exposed girls (chi square = 1.76, *p* = .184). We also found no significant gender differences in related versus unrelated offspring as described in Table 2 in our article. In response to Obel *et al.* (2), we have now explored associations between smoking in pregnancy by gender. In the unrelated group (104 unexposed boys, 2 exposed boys, 106 unexposed girls, 7 exposed girls; 2 unexposed with missing gender), there is no significant association between smoking in pregnancy and ADHD in either boys (β = −.114, *p* = .246) or girls (β = .010, *p* = .914). In the related group (262 unexposed boys, 26 exposed boys, 256 unexposed girls, 11 exposed girls), there is significant association between smoking in pregnancy in boys (β = .127, *p* = .031) but not for girls (β = .032, *p* = .601). Gender differences in the association between smoking in pregnancy and offspring ADHD have not been consistently reported (2,3). It may be that the association (and inherited effect) is stronger in boys, but by subdividing our sample into such small numbers, we believe it is not safe to draw firm conclusions. However, we agree gender effects could be usefully examined in the future.

On a separate note, the authors wonder if the birth weight difference between related and unrelated groups is an error. Although the mean birth weight appears higher in related children, this is not an error and the difference in birth weight for the related and unrelated groups is not statistically significant (4). We have also checked the birth weight means reported in the text for those exposed and not exposed to smoking in pregnancy. These too are correct. In addition, as we have previously reported, the same pattern of results is observed between smoking in pregnancy and birth weight when only singleton births are included and when statistically adjusting for gestational age (5).

We are pleased that the authors find our design interesting. As highlighted in our original article, all designs have limitations and our sample size for those exposed is small. We believe it is important to consider our results in the context of those from other designs (animal studies, siblings discordant for exposure to smoking, and children of twin studies) as discussed in our article. Taken together, the pattern of findings suggests that the previously observed association between maternal smoking and ADHD might represent an inherited/familial confound. We also suggest it is important to consider the possibility that associations observed in epidemiological studies could arise from unmeasured confounds including inherited ones.
